# Engineered *Lactococcus lactis* Secreting IL-23 Receptor-Targeted REX Protein Blockers for Modulation of IL-23/Th17-Mediated Inflammation

**DOI:** 10.3390/microorganisms7050152

**Published:** 2019-05-27

**Authors:** Tina Vida Plavec, Milan Kuchař, Anja Benko, Veronika Lišková, Jiří Černý, Aleš Berlec, Petr Malý

**Affiliations:** 1 Department of Biotechnology, Jožef Stefan Institute, Jamova 39, SI-1000 Ljubljana, Slovenia; tina.plavec@ijs.si (T.V.P.); benko.anja@gmail.com (A.B.); 2 Faculty of Pharmacy, University of Ljubljana, Aškerčeva 7, SI-1000 Ljubljana, Slovenia; 3 Laboratory of Ligand Engineering, Institute of Biotechnology of the Czech Academy of Sciences, v. v. i., BIOCEV Research Center, Průmyslová 595, 252 50 Vestec, Czech Republic; milan.kuchar@ibt.cas.cz (M.K.); veronika.liskova@ibt.cas.cz (V.L.); 4 Laboratory of Structural Bioinformatics of Proteins, Institute of Biotechnology of the Czech Academy of Sciences, v. v. i., BIOCEV Research Center, Průmyslová 595, 252 50 Vestec, Czech Republic; jiri.cerny@ibt.cas.cz

**Keywords:** lactococcus, binding protein, albumin-binding domain, IL-23 cytokine, IL-23R, surface display

## Abstract

*Lactococcus lactis*, a probiotic bacterium of food origin, has recently been demonstrated as a suitable strain for the production and in vivo delivery of therapeutically important proteins into the gut. We aimed to engineer recombinant *L. lactis* cells producing/secreting REX binding proteins that have been described as IL-23 receptor (IL-23R) blockers and IL-23R antagonists suppressing the secretion of cytokine IL-17A, a pivotal step in the T-helper Th17-mediated pro-inflammatory cascade, as well as in the development of autoimmune diseases, including inflammatory bowel disease (IBD). To reach this goal, we introduced cDNA sequences coding for REX009, REX115, and REX125 proteins into plasmid vectors carrying a Usp45 secretion signal, a FLAG tag sequence consensus, and a LysM-containing cA surface anchor (AcmA), thus allowing cell–surface peptidoglycan anchoring. These plasmids, or their non-FLAG/non-AcmA versions, were introduced into *L. lactis* host cells, thus generating unique recombinant *L. lactis*–REX strains. We demonstrate that all three REX proteins are expressed in *L. lactis* cells and are efficiently displayed on the bacterial surface, as tested by flow cytometry using an anti-FLAG antibody conjugate. Upon 10-fold concentration of the conditioned media, a REX125 secretory variant can be detected by Western blotting. To confirm that the FLAG/non-FLAG REX proteins displayed by *L. lactis* retain their binding specificity, cell-surface interactions of REX proteins with an IL-23R-IgG chimera were demonstrated by flow cytometry. In addition, statistically significant binding of secreted REX009 and REX115 proteins to bacterially produced, soluble human IL-23R was confirmed by ELISA. We conclude that REX-secreting *L. lactis* strains were engineered that might serve as IL-23/IL-23R blockers in an experimentally induced mouse model of colitis.

## 1. Introduction

Inflammatory bowel disease (IBD) is a debilitating chronic inflammation of the gastrointestinal (GI) tract. It can manifest itself either as Crohn’s disease (CD) or ulcerative colitis (UC). CD can affect any part of the GI tract. It involves submucosal and mucosal layers, leading to bleeding, abdominal pain, diarrhea, and malnourishment. In UC, the inflammation is limited to the colon [1]. The exact aetiology of IBD is not known, but is characterized by the disturbance of immune control over gut microbiota [1]. Immunological disturbance is caused by an imbalance between regulatory and pro-inflammatory T cells. T helper Th17 cells are characteristically increased in IBD and contribute to inflammation by producing IL-17, a key pro-inflammatory cytokine of IBD [2]. The expansion and maintenance of Th17 cells is promoted by IL-23, and the whole cascade is referred to as the IL-23/IL-17 axis. The importance of the latter was substantiated by animal trials, in which the inactivation of certain components of the axis protected against IBD [1]. Also, in patients with IBD, levels of IL-23 and Th17 cell cytokines were increased in the intestinal mucosa, plasma, and serum [3].

IL-23 is a composite cytokine composed of the cytokine subunits IL-23p19 and p40. The latter subunit is shared with IL-12. Similarly, the receptor for IL-23 is also composed of two subunits, IL-23R and IL-12Rb1, the latter again being shared with a receptor for IL-12 [4]. Binding of IL-23 leads to dimerization of IL-23R and IL-12Rb1, induction of JAK2 tyrosine kinase, STAT3 phosphorylation, and transcription of inflammatory genes. IL-23 is expressed by macrophages, dendritic cells, neutrophils, and epithelial cells [3].

Blocking of IL-23/IL-23R signaling is suggested as a valid therapeutic strategy for IBD [5]. The monoclonal antibody ustekinumab that blocks the p40 subunit, which is common to IL-12 and IL-23 cytokines, was recently approved for the treatment of CD [6]. Monoclonal antibodies specific for IL-23 recognize the p19 subunit, and include risankizumab, brazikumab, and mirikizumab [5], which are all in various phases of clinical trials [5]. The targeting of IL-23R is somewhat less developed. A small IL-23R-specific antagonist was developed by designing a D-peptide on the basis of flexible regions of IL-23R [7]. A non-immunoglobulin polypeptide binder of IL-23R on the basis of an atrimer scaffold has been reported [8]. In our group, a series of IL-23R protein binders was recently developed, based on a scaffold of an albumin binding domain (ABD). Variants of binders were termed REX, and were capable of inhibiting an IL-23-dependent, ex vivo expansion of IL-17-producing cells [9].

ABDs are one of more than 20 different types of non-immunoglobulin scaffolds that have been selected against more than 100 targets [10]. They have several advantages over antibodies, including small size, stability, robustness, and a monomeric structure, as well as a lack of disulfide bonds that facilitates their expression in bacteria [10]. A highly complex combinatorial library of ABD variants was selected, using ribosome display, against various targets.

REX binders were targeted to recombinant extracellular domains of human IL-23R [9], ARS binding proteins were selected against recombinant extracellular moiety of human IL-17RA [11], and ILP binders were targeted to recombinant fusion protein p19/IL-23 with a maltose binding protein [12]. For the selection of PAB proteins, a bacterial product of human prostate secretory protein 94 (PSP94) was used [13]. A bacterially-produced B subunit of shiga toxin 1 was assembled for the selection of shiga toxin-specific binders [14], and the bacterial product of human interferon gamma (IFNγ) was used for the generation of binders of IFNγ [15].

Lactic acid bacteria are Gram-positive bacteria, mainly from genera *Lactobacillus* and *Lactococcus.* They are safe, and have been used as part of human nutrition for centuries [16]. They form part of human intestinal and vaginal microbiota, and several of them have confirmed beneficial health properties and are used as probiotics [17]. Their health-promoting activity can be improved by genetic engineering, and they can be applied as a live delivery vehicle for therapeutic proteins to mucosal surfaces [18]. For the alleviation of the symptoms of IBD, our group developed a recombinant *Lactococcus lactis* capable of displaying a tumor necrosis factor alpha (TNFα)-binding protein on its surface, resulting in the effective removal of TNFα from the solution [19]. The effectiveness of recombinant *L. lactis* was established in animal [20] as well as in an ex vivo tissue model [21].

Recently, we have effectively displayed ABD-based ILP binders of IL-23 on the surface of *L. lactis* [12]. This work has been upgraded in the present work, which describes the secretion of IL-23R binders from *L. lactis*. A combination of both approaches would simultaneously target two elements of the IL-23/IL-17 axis, and therefore holds great potential for the synergistic treatment of IBD.

## 2. Materials and Methods

### 2.1. Bacterial Strains, Media, and Growth Conditions

The bacterial strains used in this study are shown in Table 1. *Lactococcus lactis* NZ9000 was grown at 30 °C in M17 medium (Merck, Darmstadt, Germany), supplemented with 0.5% glucose (GM-17) without agitation or in the same medium solidified with 1.5% agar. Electroporation of *L. lactis* was performed according to [22], using a Gene Pulser II apparatus (Bio-Rad, Hercules, CA, USA). To maintain selection pressure on transformation, 10 µg/mL chloramphenicol was added to the growth medium. *E. coli* strain DH5α was grown at 37 °C with agitation in a lysogeny broth (LB) medium supplemented with 100 µg/mL ampicillin.

### 2.2. DNA Manipulation and Plasmid Construction

Restriction enzymes and T4 DNA ligase were purchased from Thermo Scientific. PCR amplifications were performed with Dream Taq or Phusion Hot Start polymerase (Thermo Fisher Scientific, Waltham, MA, USA), according to the manufacturer´s protocols. PCR products were routinely ligated to pGEM-T Easy (Promega, Madison, WI, USA) or pJET1.2 (CloneJET PCR Cloning Kit, Thermo Fisher Scientific, Waltham, MA, USA) for sequencing and further cloning. Plasmid DNA was isolated with a NucleoSpin Plasmid (Macherey-Nagel, Duren, Germany), with an additional lysozyme treatment step in the case of *L. lactis*. Nucleotide sequencing was performed by GATC-Eurofins Genomics AT GmbH (Ebersberg, Germany). Primers (IDT) and plasmids are listed in Table 1.

The *rex009, rex115*, and *rex125* genes were amplified by PCR from original plasmids carrying sequences of REX009, REX115, and REX125 proteins [12], using ILP030-F/Rex009-R-Eco, ILP030-F/Rex115-R-Eco, and ILP030-F/Rex125-R-Eco primer pairs, respectively. All amplicons were cloned to a pSDLBA3b plasmid for surface display [19] via BamHI/EcoRI restriction sites (yielding pSD-REX009, pSD-REX115, and pSD-REX125). Similarly, genes were amplified using primer pairs ILP030-F/Rex009-R-Xba, ILP030-F/Rex115-R-Xba, and ILP030-F/Rex125-R-Xba, and cloned to a pSDLBA3b plasmid via BamHI/XbaI, thereby yielding a pSC-REX series (pSC-REX009, pSC-REX115, and pSC-REX125) that enables secretion. To insert a FLAG-tag nucleotide sequence upstream of *rex* sequences in all pSD-REX and pSC-REX plasmids, the *usp45* sequence was amplified from pSDLBA3b using primers Usp1-NcoI/FLAG_Bam_R, digested with NcoI/BamHI, and cloned into equally treated plasmids. This yielded plasmids pSD-REX009-FLAG, pSD-REX115-FLAG, pSD-REX125-FLAG, pSC-REX009-FLAG, pSC-REX115-FLAG, and pSC-REX125-FLAG.

### 2.3. Expression of IL-23R Binding Fusion Proteins in L. lactis

Overnight cultures of *L. lactis* NZ9000 transformed with the appropriate plasmid (pSD-REX009, pSD-REX115, pSD-REX125, pSC-REX009, pSC-REX115, pSC-REX125, pSD-REX009-FLAG, pSD-REX115-FLAG, pSD-REX125-FLAG, pSC-REX009-FLAG, pSC-REX115-FLAG, pSC-REX125-FLAG, or pNZ8148—an empty plasmid control) were diluted (1:100) in 10 mL or 50 mL of fresh GM-17 medium, and grown to optical density A_600_ = 0.80. Fusion protein expression was induced with 25 ng/mL nisin (Fluka Chemie AG, Buchs, Switzerland). After three hours of incubation, 1 mL of culture was stored at 4 °C for flow cytometry, and the remaining cell culture was centrifuged at 5000× *g* for 10 min. The cell pellet was resuspended in 400 µL of phosphate-buffered saline (PBS, pH 7.4) and stored at –20 °C for SDS-PAGE. Media were filtered through sterile 0.22 µm pore filters (Sartorius, Göttingen, Germany) and concentrated 10-fold using 1K Microsep Advance Centrifugal Devices (Pall, Port Washington, NY, USA).

### 2.4. SDS-PAGE and Western Blot

SDS-PAGE was performed with a Mini-Protean II apparatus (Bio-Rad, Hercules, CA, USA). Samples were thawed in an ice bath, briefly sonicated with a UPS200S sonicator (Hielscher Ultrasonics, Teltow, Germany), mixed with 2x Laemmli Sample buffer and dithiothreitol, and denatured by heating at 100 °C before loading. Page Ruler Plus pre-stained standard (Hielscher Ultrasonics, Teltow, Germany) was used for molecular weight comparison. Proteins were transferred to a nitrocellulose membrane (GE Healthcare Life Sciences, Marlborough, MA, USA) using wet transfer at 100 V for 90 minutes. Membranes were blocked in 5% non-fat dried milk in TBST (50 mM Tris-HCl, 150 mM NaCl, 0.05% Tween 20, pH 7.5) and incubated overnight at 4 °C with FLAG-tag Rabbit Polyclonal Antibody (Proteintech, Rosemont, IL, USA; 1:1000) in 5% non-fat dried milk in TBST. Following three washes with TBST, membranes were incubated for 2 h with goat anti-rabbit IgG, Dylight 650 conjugate (Thermo Fisher Scientific, Waltham, MA, USA; 1:10000), or horseradish peroxidase-conjugated goat anti-rabbit antibody (Jackson ImmunoResearch Europe, Ely, United Kingdom; 1:10000) in 5% non-fat dried milk in TBST. After three further washes with TBST, images were acquired using a ChemiDoc MP Imaging System (Bio-Rad, Hercules, CA, USA) using fluorescence or chemiluminescence (following incubation with Lumi-Light reagent (Roche, Basel, Switzerland), respectively.

### 2.5. Flow Cytometry

For flow cytometry, 20 µL of a cell culture in the stationary phase was added to 500 µL of Tris-buffered saline (PBS; 50 mM Phosphate buffer, 150 mM NaCl, pH 7.5) and centrifuged for 5 min at 5000× *g* and 4 °C. The pellet was resuspended in 200 µL of recombinant human IL-23R Fc chimera (1400-IR, R&D Systems, 1 µg/mL in PBS). After 2 h of incubation at room temperature (RT) with constant shaking at 100 rpm, cells were washed three times with 200 µL 0.1% PBS-Tween (PBST) and resuspended in 200 μl of PBS with goat anti-human IgG (Fc specific; BioLegend, San Diego, CA, USA; diluted 1:2000). After 2 h of incubation at RT with constant shaking at 100 rpm, cells were washed three times with 200 µL 0.1% PBST and resuspended in 200 μL of PBST with Alexa Fluor 488-conjugated donkey anti-goat IgG (Invitrogen, Carlsbad, CA, USA; diluted 1:2500). 2 h incubation at RT was repeated, cells were washed again three times with 200 μL PBST, and finally were resuspended in 500 µL PBS. Samples were analysed with a FACSCalibur flow cytometer (Becton Dickinson, Franklin Lakes, NJ, USA) using excitation at 488 nm and emission at 530 nm in the FL1 channel. Bacterial cells were gated in an Side Scatter–Forward Scatter (SSC-FSC) plot, as depicted in Figure S1, for a representative experiment. The results are presented as mean fluorescence intensity (MFI) values of at least 20,000 lactococcal cells. The result was expressed as the average of at least three independent experiments.

A similar protocol was applied for the detection of FLAG-tagged ILP fusion proteins. FLAG-tag Rabbit Polyclonal Antibody (Proteintech Group, Chicago, IL, USA; diluted 1:500) was used as a primary antibody and anti-rabbit IgG Fab2 Alexa Fluor 488 (Molecular Probes, Eugene, OR, USA; diluted 1:2000) was used as a secondary antibody.

### 2.6. Production of the Recombinant IL-23 Receptor

The extracellular part of the IL-23 receptor (IL-23Rex, amino acids 24–350) coded by plasmid DNA (His_6_–IL-23Rex–pET-28b [9] was produced in *E. coli* SHuffle strain (SHuffle T7 Express Competent *E. coli*, New England Biolabs, Ipswich, MA, USA). Bacterial cells were grown in LB broth with kanamycin (60 µg/mL) at 30 °C; protein production was induced by adding 1 mM isopropyl β-D-1-thiogalactopyranoside (IPTG) after the culture reached the density OD_600_ = 0.8, and the cells were cultivated overnight at 23 °C. The next day, the culture was collected by centrifugation and sonicated in TN buffer (50 mM Tris, 300 mM NaCl, pH = 8.0), after which the disrupted cells were spun down at 40,000 g for 20 min and supernatant was used for the purification of soluble protein using Ni-NTA agarose.

### 2.7. IL-23R Binding Assay

A polysorp plate (NUNC, Roskilde, Denmark) was coated by IL-23Rex recombinant protein (10 µg/mL) in a coating buffer (100 mM bicarbonate/carbonate solution, pH = 9.6) at 7 °C overnight. The next day, the plate was washed by PBST (PBS buffer containing 0.05% Tween, pH = 7.4) and blocked by PBSTB (1% bovine serum albumin (BSA) in PBST). The medium samples containing REX protein variants secreted by *L. lactis*, as well as purified biotinylated REX115-TolA protein [9] diluted in PBSTB as a positive control, were both applied. REX variant binding to IL-23Rex was detected by an anti-DDDDK tag antibody [M2] (horseradish peroxidase (HRP)) (abcam, Cambridge, United Kingdom) diluted in PBSTB 1:20,000, and bound REX115-TolA protein was detected using a streptavidin-HRP conjugate (Pierce, Rockford, IL, USA) diluted in the same buffer at 1:10,000. The result was visualized by the enzymatic reaction of HRP with TMB-Complete 2 substrate (TestLine Clinical Diagnostics s.r.o., Czech Republic); the reaction was stopped by 2 M sulfuric acid, and absorbance at 450 nm was measured.

### 2.8. Modeling of IL-23R/REX Interaction by Docking

Models of studied ABD variants (REX115 and REX125) were built using the MODELLER 9v14 suite of programs [26,27] based on the ABDwt structure (pdb id 1gjt) [28]. The model of murine IL-23R was obtained by MODELLER from the available crystal structure of human IL-23/IL-23R complex (pdb id 5mzv) [29], using the pdb chain C and a pairwise sequence alignment of IL-23R proteins. Amino acid sequences of human IL-23R (uniprot Q5VWK5) and murine IL-23R (uniprot Q8K4B4) corresponding to the crystallized extracellular part of the receptor (residues 24-330) were aligned with the Clustal Omega program [30]. The proteins share 78% identity and 95% sequence similarity in this region. The flexible side chain protein–protein docking of ABD variants to the murine IL-23R was performed using a local copy of the ClusPro server [31,32].

### 2.9. Statistics

Data were analyzed with GraphPad Prism 5.0 software. Mean fluorescence intensities (MFIs), obtained by flow cytometry, were compared to the control using a *t*-test. Significant differences (*p* < 0.05) were highlighted. Binding of REX binders to immobilized IL-23R, determined by ELISA, was compared by ANOVA. Significant differences (*p* < 0.05) were highlighted.

## 3. Results

### 3.1. Genetic Constructs for the Display and Secretion of REX Binders of IL-23R

**Figure 1:**
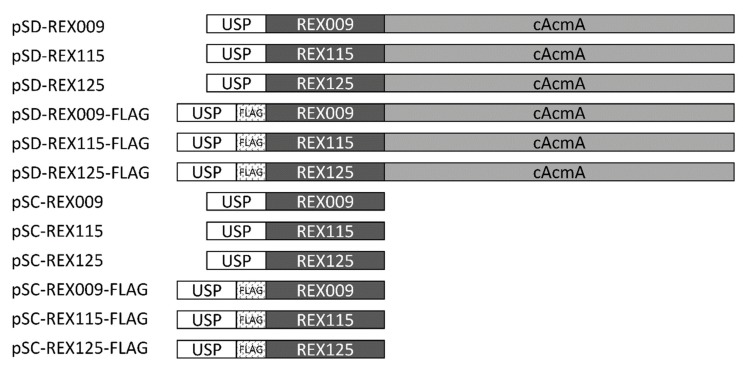
Scheme of genetic constructs for display (pSD) and secretion (pSC) of REX binders.

Three previously selected REX binders of IL-23R (REX009, REX115, REX125) [9] were considered in the present study. Genes for REX binders were fused with a Usp45 secretion signal, and optionally with a cAcmA surface anchor [19,33] or a FLAG-tag for detection. This resulted in 12 genetic constructs that are schematically depicted in Figure 1.

### 3.2. Expression of REX Fusion Protein in L. lactis

**Figure 2:**
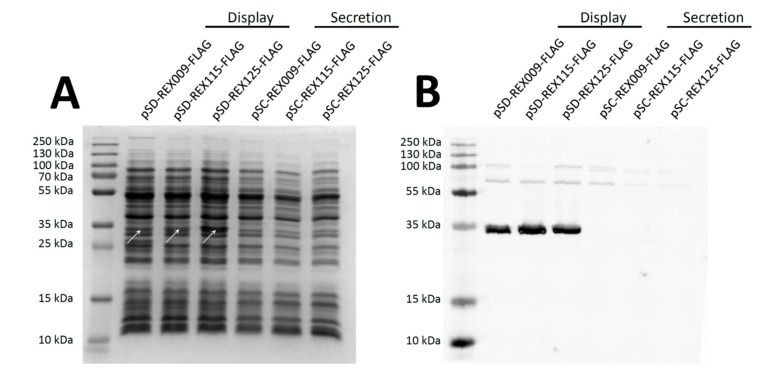
Detection of FLAG-tagged REX fusion proteins in the cell lysate of *L. lactis*. (**A**) SDS-PAGE stained with Coomassie Brilliant Blue. (**B**) Western blotting with an anti-FLAG-tag antibody. REX proteins are in fusion with the Usp45 secretion signal (Secretion), or with the Usp45 secretion signal and a cAcmA surface anchor (Display).

FLAG-tagged REX fusion proteins were expressed under the control of the inducible nisin promoter [23], and secreted from cells with a Usp45 secretion signal [34]. In the total cell lysate (including cell wall debris), REX variants that contained a cAcmA anchor (those displayed on the bacterial surface), were detected with both Coomassie staining (Figure 2A) and Western blotting (Figure 2B). According to expectations, secreted variants without the cAcmA anchor were not detected in cell lysates.

**Figure 3:**
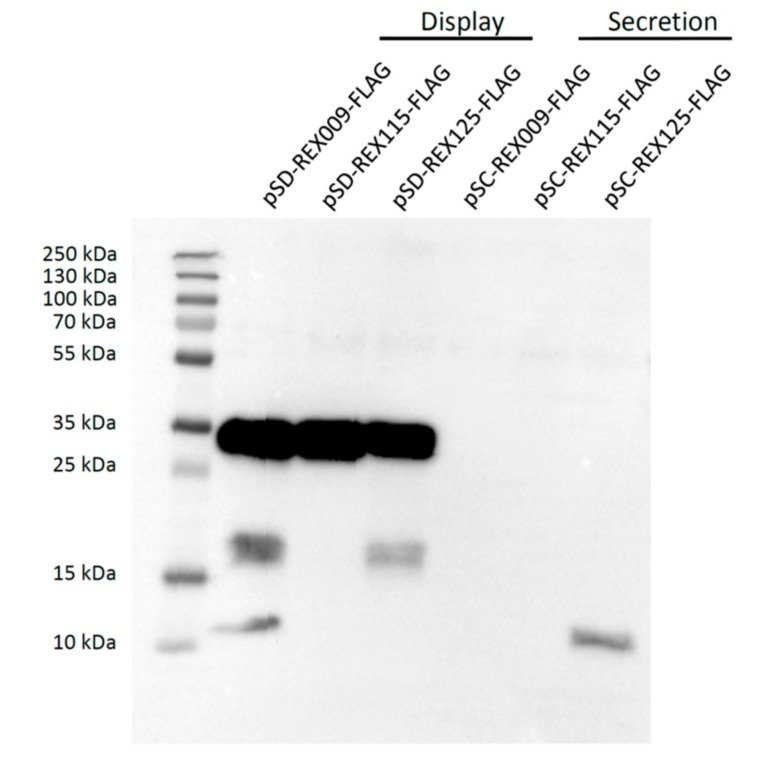
Western blot of 10-fold concentrated conditioned media of *L. lactis* cultures expressing FLAG-tagged REX variants.

To detect secreted REX fusion proteins in the conditioned growth media, the latter were concentrated 10-fold and analysed with Western blotting (Figure 3). At this stage, cAcmA anchor-containing fusion proteins were detected in considerable amounts, confirming previous observations that not all secreted anchor-containing fusion proteins are actually attached to the bacterial surface [35]. Some degradation products were observed with REX009 and REX125 fusion proteins. On the other hand, among secreted fusion proteins without a surface anchor, only REX125 was detected in the conditioned medium.

### 3.3. Display of REX Fusion Proteins on the Surface of L. lactis

**Figure 4:**
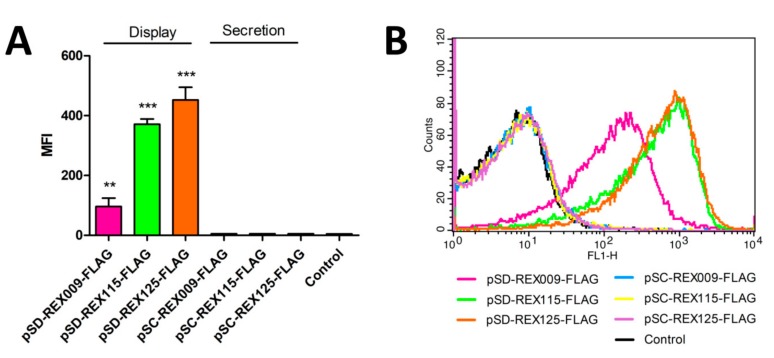
Flow cytometry of *L. lactis* cells displaying or secreting FLAG-tagged REX variants, detected with an anti-FLAG antibody. Control: *L. lactis* containing an empty plasmid of pNZ8148. (**A**) Mean fluorescence intensity (MFI) or (**B**) the shift in fluorescence intensity of representative measurements are depicted. Error bars denote standard deviations. Significant differences (** *p* < 0.005, *** *p* < 0.001; Student’s *t*-test) are marked with an asterisk.

All three REX fusion proteins containing cAcmA anchor were significantly displayed on the bacterial surface, as assessed with flow cytometry, using anti-FLAG antibodies (Figure 4). The largest extent of surface display was achieved with REX125 fusion protein, followed by REX115 fusion protein. The lowest extent of the surface display was observed with REX009 fusion protein. According to expectations, no surface display was observed with secreted REX variants without the cAcmA anchor.

### 3.4. Binding of IL-23R-Fc Chimera to the Surface of REX-Displaying L. lactis

**Figure 5:**
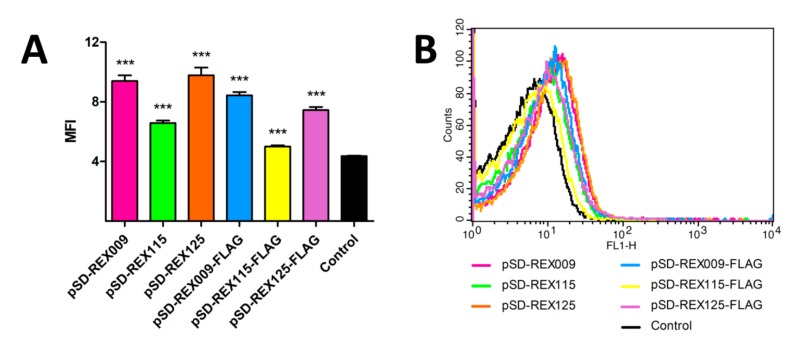
Flow cytometry of *L. lactis* cells displaying or secreting FLAG-tagged REX variants, detected with IL-23R-Fc fusion proteins. Control: *L. lactis* containing an empty plasmid of pNZ8148. (**A**) Mean fluorescence intensity (MFI) or (**B**) a shift in fluorescence intensity of representative measurements are depicted. Error bars denote standard deviations. Significant differences *** *p* < 0.001; Student’s *t*-test) are marked with an asterisk.

The functionality of REX variants displayed on the bacterial surface was assessed by their ability to bind a recombinant human IL-23 receptor fused to the Fc region of human IgG. This form of the receptor–IgG chimera was employed for specific detection using flow cytometry. REX009 and REX125 binders exhibited a similar ability to bind IL-23R-Fc, while the binding with REX115 was lower (Figure 5). REX variants with a FLAG-tag exhibited slightly lower binding in comparison to variants without a FLAG-tag.

### 3.5. Binding of L. lactis-Secreted REX Variants to Recombinant IL-23R in ELISA

**Figure 6:**
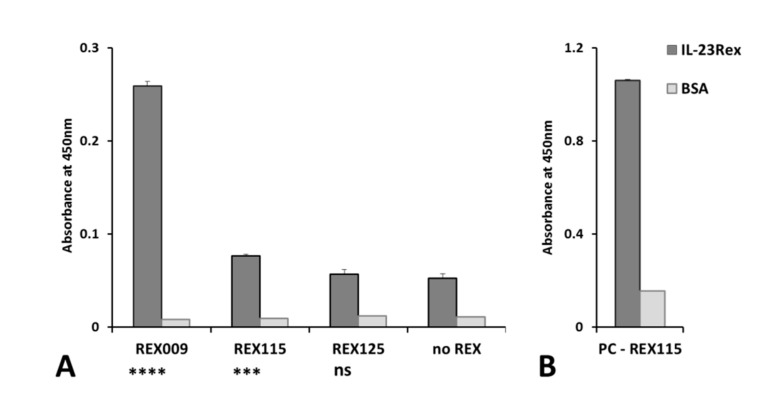
Binding of secreted REX variants in ELISA. The 96-well plate was coated with recombinant IL-23Rex protein, and samples of the medium with secreted REX proteins were applied (**A**). Purified protein REX115–TolA diluted in PBSTB was used as a positive control (**B**). The binding of REX proteins to IL-23Rex, and to BSA as a negative control, was detected by anti-DDDDK tag antibody [M2] conjugated with horseradish peroxidase (HRP) (A), or by streptavidin with HRP (B). Each bar represents the mean value with standard deviation and significant differences between mean values of the negative control (no REX), and the samples were analyzed by ANOVA (*** *p* < 0.004, **** *p* < 0.001, ns: no significant difference).

The presence of secreted REX protein variants in medium of particular recombinant REX-containing *L. lactis* strains was verified by ELISA. To this goal, a bacterially produced extracellular moiety of human IL-23 receptor was used. Non-concentrated samples of media were tested for the binding of REX proteins to an immobilized, 327 amino acid IL-23Rex [9]. The results shown in Figure 6A confirm statistically significant binding of REX009 and REX115 variants in comparison to a control non-REX *L. lactis* medium. The validity of the used detection method was demonstrated using a purified in vivo biotinylated 38 kDa REX115–TolA–Avi protein binder [9] coated in PBS buffer at a concentration of 1 µg/mL, as shown in Figure 6B.

## 4. Discussion

The composition of intestinal microbiota in IBD is considerably altered; the latter disease can therefore be considered a dysbiosis. The characteristic microbial fingerprint of IBD microbiota consists of an increase in phylum Bacteroidetes and family *Enterobacteriaceae*, as well as a decrease in the phylum Firmicutes [36]. The change in microbiota composition results in different metabolic potential of microbiota, which is exemplified by the decreased production of short-chain fatty acids—e.g., butyric acid, which represents an important energy source for epithelial cells [37]. The composition of microbiota could be influenced by food management and prebiotic supplementation [37]; alternatively, supplementation with probiotic lactic acid bacteria that belong to the phylum Firmicutes was also established as beneficial [38]. An additional benefit of the latter approach could be achieved with genetic engineering of the bacteria, as suggested in the present study.

ABD-based REX binders REX009, REX115, and REX125 were selected as the most promising binders of IL-23R in our previous study [9]. All three of them were, therefore, included in the present study of the delivery of REX binders by safe lactic acid bacterium *L. lactis*. Two types of genetic constructs were prepared, enabling either secretion (pSC plasmid series), or surface display (pSD plasmid series) of REX binders. The secretion of REX binders was intended for the neutralization of IL-23R, and consequently, interruption of the IL-23/IL-17 axis. The display of REX binders could, on the other hand, lead to the targeting of IL-23R-expressing cells by recombinant bacteria. Additionally, the surface display of REX binders facilitates testing of the expression level and functionality of REX binders with the use of flow cytometry.

The secretion of REX binders was achieved by fusing them with a Usp45 secretion signal. For surface display, REX binders were fused with the Usp45 secretion signal and a surface anchor from the endogenous AcmA protein. All three fusion proteins for surface display were detected in cell lysates with both Coomassie Brilliant Blue staining and Western blotting. Secreted proteins could not be detected in the cell lysate, possibly due to their lower molecular weight (8 kDa, compared to 30.5 kDa for fusion proteins for surface display) that led to their more effective secretion from the cells.

In the conditioned media, the REX binders were present in relatively large volume. These media were, therefore, concentrated 10-fold. However, only REX125 could be detected, indicating its highest level of secretion. Surface display of all three REX binders was confirmed via FLAG-tag, using flow cytometry. REX125 again had the highest surface display, corroborating it having highest level of secretion, as observed in the conditioned media. In contrast, REX009 had the lowest surface display, or its conformation partially obstructed the FLAG-tag.

The surface display of REX binders, as well as their binding capability, was evaluated via their ability to bind soluble IL-23R in the form of a secreted IgG chimera. REX125 again had a high binding ability comparable to that of the REX009. FLAG-tagged fusion proteins demonstrated a slightly lower IL-23R binding signal, probably due to steric hindrances.

The binding of REX proteins secreted by particular recombinant *L. lactis* strains was tested in ELISA. REX009 and REX115 protein variants present in conditioned media confirmed a statistically significant binding to the immobilized recombinant IL-23R, prepared in the form of bacterially produced, soluble human extracellular protein.

Based on data obtained by flow cytometry and ELISA experiments, REX009 seems to be the most appropriate variant for further in vivo experiments. REX125 is well expressed and displayed by *L. lactis* cells, and in addition, significantly binds the IL-23R –IgG chimera. However, as a secreted protein, REX125 exhibits the lowest binding signal to a coated, soluble IL-23R in ELISA. As this protein is secreted to the medium (Figure 4), the loss of function might be rather attributed to a different conformation or changes in the IL-23R binding mode compared to the AcmA-oriented, displayed REX variant interacting with the IL-23R–IgG chimera.

**Figure 7:**
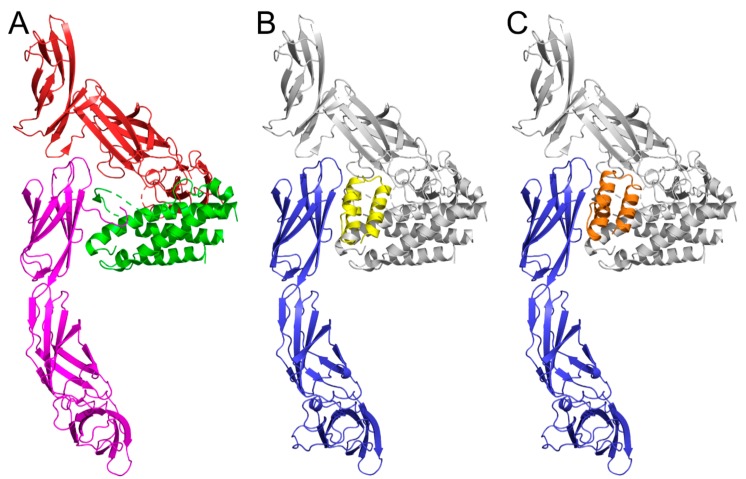
Predicted binding mode of ABD variants to the IL-23R. (**A**) The crystal structure of human IL-23/IL-23R complex (pdb id 5mzv) [29] showing the IL-23R in magenta, the IL-23 subunit p19 in green, and the IL-12 subunit p40 of IL23 in red. (**B**) The most probable predicted binding mode of the REX115 (yellow) to the murine IL-23 model (blue) overlapping with the position of IL-23 (grey) from the human IL-23/IL-23R complex. (**C**) The most probable predicted binding mode of the REX125 (orange) to the murine IL-23 model (blue).

REX protein binding variants were originally identified as binders of the human IL-23 receptor. As we intended to use the generated REX-secreting *L. lactis* recombinant strains for in vivo testing, as antagonists of the IL-23R in the mouse model of experimentally induced colitis, we generated a homology model of the murine IL-23R, based on the crystal structure of the human IL-23/IL-23R complex available in the PDB database, and tested the binding ability of particular REX variants by docking. The most probable binding modes predicted for REX115 and REX125 variants, shown in Figure 7, suggest IL-23R blocking potential in mice. For REX009, the used docking approach did not predict similar binding modes, potentially interfering with mouse IL-23 binding. This might be due to a possible contribution of the N-terminal region of the IL-23R to the binding, similar to interactions in the human IL-23/IL-23R complex (Figure 7A). However, all three REX variants bind to IL-23R-expressing mouse NIH-3T3 fibroblasts, as tested by flow cytometry (Figure S2). In addition, REX115 protein binds to these cells with an extraordinarily high binding affinity, estimated by a LigandTracer-mediated measurement to be 0.1 nM. Interestingly, the binding affinity of this protein to green monkey COS-7 kidney cells that express monkey IL-23R (Figure S3), which is sequentially almost identical to the human receptor, is 1 nM (Figure S4), a value comparable to that found for REX009 IL-23R binding [9] measured by surface plasmon resonance.

In the present study, we observed that the sequence of ABD variants (e.g., of different REX binders) affects their expression, secretion, and surface display, as well as their conformation in *L. lactis*. Our data demonstrate that all of the used REX proteins secreted by the *L. lactis* cells bind human IL-23R and suggest binding to the mouse receptor, making them suitable candidates for further testing in an IBD mouse model, preferably together with our previously developed *L. lactis* displaying IL-23 binding ILP proteins [25]. Planned animal experiments similar to the one we already reported [20] will help to overcome the limitations of in vitro modelling of cytokine binding presented in the present study, and will provide necessary data on the physiological effects of engineered bacteria.

## Figures and Tables

**Table 1:** Strains, plasmids, and primers used in the study. Restriction recognition sites are underlined.

Strain, Plasmid, or Gene	Relevant Features or Sequence (5’–3’)	Ref. or Source
**Strain**		
*E. coli*		
DH5α	endA1 glnV44 thi-1 recA1 relA1 gyrA96 deoR F- Φ80d*lacZ*ΔM15 Δ(*lacZYA-argF*)U169, hsdR17(rK- mK+), λ–	Invitrogen
TOP10	F– mcrA Δ(mrr-hsdRMS-mcrBC) Φ80lacZΔM15 ΔlacX74 recA1 araD139 Δ(ara leu) 7697 galU galK rpsL (StrR) endA1 nupG	Life technologies
BL21 λ(D3)	E. coli B F – dcm ompT hsdS (rB– mB–) gal λ(DE3)	[15]
*L. lactis*		
NZ9000	MG1363 *nisRK* Δ*pepN*	[23]
**Plasmid**		
pNZ8148	pSH71 derivative, P*nisA,* CmR, nisin-controlled expression	[23]
pSDBA3b	pNZ8148 containing gene fusion of Usp45 signal peptide, B domain, and cA	[24]
pET-REX009	pET28b containing a fusion gene of REX009, tolA protein, and AviTag consensus	[9]
pET-REX115	pET28b containing a fusion gene of REX115, tolA protein, and AviTag consensus	[9]
pET-REX125	pET28b containing a fusion gene of REX125, tolA protein, and AviTag consensus	[9]
pSD-REX009	pNZ8148 containing gene fusion of Usp45 signal peptide, REX009, and cA	This work
pSD-REX115	pNZ8148 containing gene fusion of Usp45 signal peptide, REX115, and cA	This work
pSD-REX125	pNZ8148 containing gene fusion of Usp45 signal peptide, REX125, and cA	This work
pSD-REX009-FLAG	pNZ8148 containing gene fusion of Usp45 signal peptide, FLAG tag, REX009, and cA	This work
pSD-REX115-FLAG	pNZ8148 containing gene fusion of Usp45 signal peptide, FLAG tag, REX115, and cA	This work
pSD-REX125-FLAG	pNZ8148 containing gene fusion of Usp45 signal peptide, FLAG tag, REX125, and cA	This work
pSC-REX009	pNZ8148 containing gene fusion of Usp45 signal peptide and REX009	This work
pSC-REX115	pNZ8148 containing gene fusion of Usp45 signal peptide and REX115	This work
pSC-REX125	pNZ8148 containing gene fusion of Usp45 signal peptide and REX125	This work
pSC-REX009-FLAG	pNZ8148 containing gene fusion of Usp45 signal peptide, FLAG tag, and REX009	This work
pSC-REX115-FLAG	pNZ8148 containing gene fusion of Usp45 signal peptide, FLAG tag, and REX115	This work
pSC-REX125-FLAG	pNZ8148 containing gene fusion of Usp45 signal peptide, FLAG tag, and REX125	This work
**Primer**		
ILP030-F	TGGATCCTTAGCTGAAGCTAAAGTC	This work
Rex009-R-Eco	AGAATTCAGGTAACGAAGCTAAAATC	This work
Rex009-R-Xba	ATCTAGAAGGTAACGAAGCTAAAATC	This work
Rex115-R-Eco	AGAATTCAAGGTAAAACAGCTAAAATCC	This work
Rex115-R-Xba	ATCTAGAAGGTAAAACAGCTAAAATCC	This work
Rex125-R-Eco	AGAATTCAAGGTAACGCAGCTAAAATAG	This work
Rex125-R-Xba	AGAATTCAGGTAACGCAGCTAAAATAG	This work
Usp1-NcoI	ATAACCATGGCTAAAAAAAAGATTATCTCAGCTATTTTAATG	[19]
FLAG_Bam_R	GGATCCTTTATCATCGTCGTCTTTATAATCAGCGTAAACACCTGACAACG	[25]

## Supplementary Materials

The following are available online at https://www.mdpi.com/2076-2607/7/5/152/s1.
