# Supplementary material for: Engineered Lactococcus lactis Secreting IL-23 Receptor-Targeted REX Protein Blockers for Modulation of IL-23/Th17-Mediated Inflammation
Source: Microorganisms. 2019 May 27;7(5):152. doi: 10.3390/microorganisms7050152 (PMC6560508; doi:10.3390/microorganisms7050152)
Supplement: Supplementary file 1 [file microorganisms-07-00152-s001.pdf]

Supplementary data

Engineered *Lactococcus lactis* secreting IL-23 receptor-targeted REX protein blockers for modulation of IL-23/Th17-mediated inflammation

Tina Vida Plavec [1,2], Milan Kuchař [3], Anja Benko [1,2], Veronika Lišková [3], Jiří Černý [4], Aleš Berlec [1,2]* and Petr Malý [3,*]

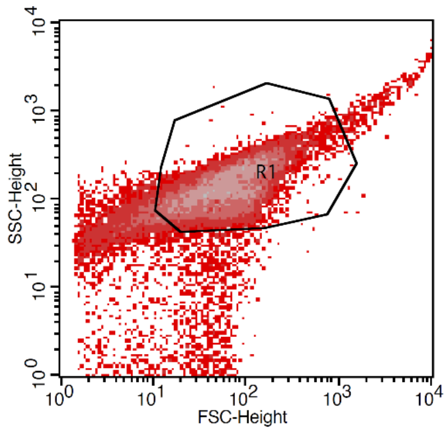

Figure S1. Representative SSC-FSC diagram for bacterial cells depicting gating strategy (R1).

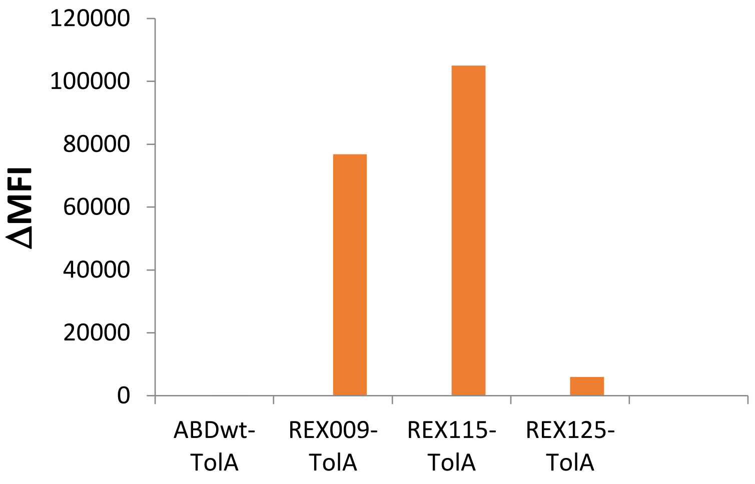

Figure S2. Binding of REX proteins to mouse NIH-3T3 fibroblasts. For the binding assay, 2.5×10^5 NIH-3T3 cells were incubated with *in vivo* biotinylated His_6-REX-TolA-AVI proteins or His_6-ABDwt-TolA-AVI negative control (10 μg/mL) for 30 min at 4 °C. The cell-bound proteins were stained with streptavidin-PE for 30 min at 4 °C and analyzed by flow cytometry.

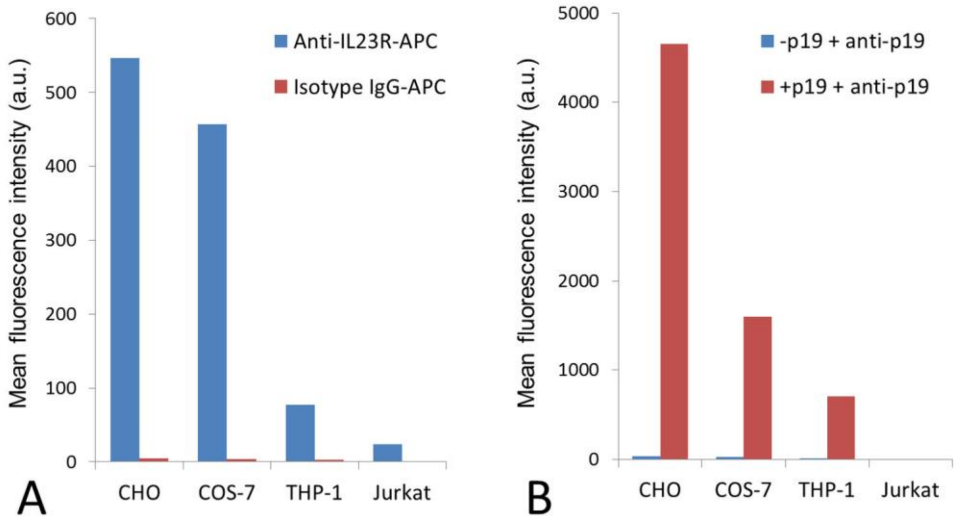

Figure S3. Expression of IL-23R on cells of selected cell lines tested by flow cytometry. For the binding assay, 2.5×10^5 cells were incubated with anti-IL23R-APC antibody conjugate (Panel A, blue columns) or isotype IgG-APC as a negative control (red columns) and analyzed by flow cytometry. Binding of recombinant p19 protein to IL-23R-expressing cells was detected by flow cytometry using a mouse anti-IL-23 mAb goat anti-mouse IgG antibody sandwich labeled with Cy5 (panel B, red columns). Staining with the secondary IgG-Cy5 conjugate only as a negative control is shown in panel B as blue columns.

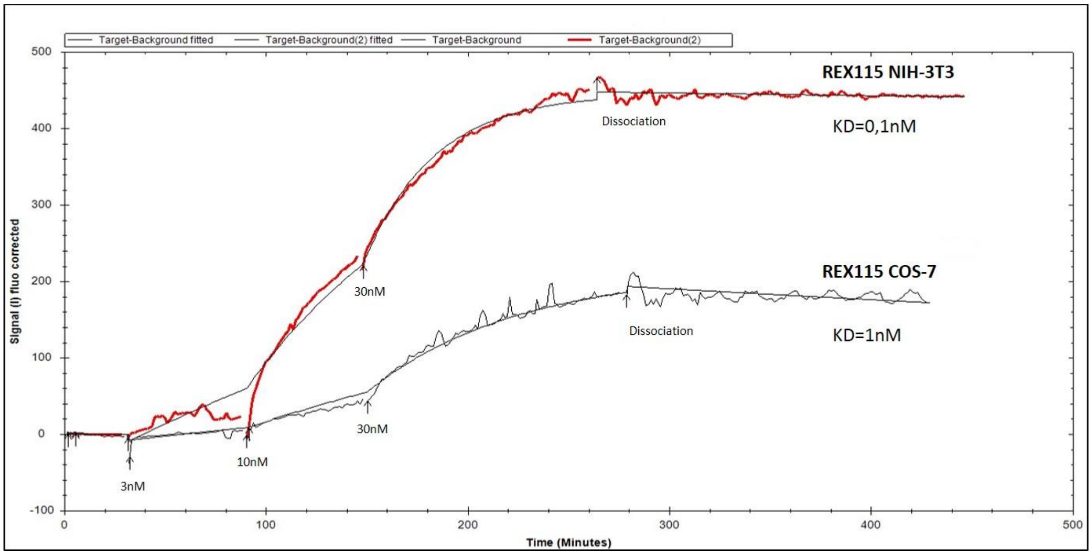

Figure S4. Binding of REX115 to mouse NIH-3T3 cells and green monkey COS-7 cells tested by LigandTracer Green Line system. For the binding assay, 10^6 cells were plated overnight on Petri dish and the next day, *in vivo* biotinylated His_6-REX115-TolA-AVI protein was added into medium and incubated gradually at three different concentrations. Cell-bound protein was stained with streptavidin-APC conjugate and the measured binding curve was analyzed using the TraceDrawer software. Analysis of the binding affinities and rate-off kinetics indicated K_d values for NIH-3T3 cells as 0.1 nM and for COS-7 cells as 1 nM.
